# Standardized Berry Extract Improves Selected Visual Function Outcomes in Presbyopia: A Randomized, Double-Blind, Placebo-Controlled Crossover Trial with Exploratory Biomarker Analysis

**DOI:** 10.3390/nu18061016

**Published:** 2026-03-23

**Authors:** Dorota Szumny, Alicja Zofia Kucharska, Karolina Czajor, Karolina Kaptsiuh, Sabina Ziółkowska, Patrycja Krzyżanowska-Berkowska, Marta Misiuk-Hojło, Monika Skrzypiec-Spring, Jakub Szyller, Adam Szeląg, Tomasz Sozański

**Affiliations:** 1 Department of Pharmacology, Faculty of Medicine, Wroclaw Medical University, Mikulicza-Radeckiego 2, 50-345 Wrocław, Poland; 2 Ophthalmology Clinic, University Clinical Hospital, Borowska 213, 50-556 Wrocław, Poland; 3 Department of Fruit, Vegetable and Plant Nutraceutical Technology, Wrocław University of Environmental and Life Sciences, Chełmońskiego 37, 51-630 Wrocław, Poland; alicja.kucharska@upwr.edu.pl (A.Z.K.);; 4 Department of Ophthlmology, Faculty of Medicine, Wroclaw Medical University, Borowska 213, 50-556 Wrocław, Poland; 5 Department of Preclinical Sciences, Pharmacology and Medical Diagnostics, Faculty of Medicine, Wrocław University of Science and Technology, Wybrzeże Wyspiańskiego 27, 50-370 Wrocław, Poland; jakub.szyller@pwr.edu.pl (J.S.);

**Keywords:** presbyopia, anthocyanins, iridoids, TRPV4, crystallins, visual function, optical coherence tomography, visual evoked potentials

## Abstract

Background/Objectives: Presbyopia is an age-related decline in near vision associated with lens stiffening and neuroretinal changes, while evidence for the effects of berry-derived phytochemicals remains limited. We investigated whether AKB, a double-standardised berry extract (anthocyanins ≥ 25%, iridoids ≥ 4.5%) from *Aronia melanocarpa*, *Lonicera caerulea*, and *Vaccinium myrtillus*, influences visual performance and circulating biomarkers potentially relevant to ocular homeostasis. Methods: In a randomised, double-blind, placebo-controlled, two-period crossover trial, 23 adults aged >50 years received AKB (400 mg twice daily) or placebo for 6 weeks, separated by a 5-week washout. Results: Compared with placebo, AKB was associated with improvements in selected visual-function outcomes, including near contrast sensitivity and visual-field parameters, together with directionally favourable changes in VEP and OCT readouts. AKB supplementation was also associated with lower circulating αA-/αB-crystallin and ALDH1A1 levels and higher circulating TRPV4 levels, whereas systemic antioxidant enzymes and advanced glycation end-products remained unchanged. Given the small sample size and the indirect nature of the biomarker assessment, these findings should be considered preliminary. Conclusions: Overall, short-term AKB supplementation was associated with modest, exploratory changes in selected functional and systemic biomarker outcomes, but larger and longer-term studies are needed to confirm clinical relevance and clarify underlying mechanisms.

## 1. Introduction

Presbyopia is an age-related decline in accommodative capacity that impairs near vision and affects an increasingly large proportion of the global population [1,2,3].

It typically becomes clinically relevant after the age of 45 and is expected to increase further as the population ages [4]. Although presbyopia is often considered a normal consequence of ageing, it has a substantial impact on daily functioning and quality of life [5]. Current management is based mainly on optical correction or surgical intervention, while pharmacological and nutritional strategies remain comparatively underexplored [6].

The pathophysiology of presbyopia is multifactorial. The condition is primarily associated with progressive loss of lens flexibility and reduced accommodative responsiveness, but age-related changes also involve long-term protein instability, altered tissue biomechanics, and impaired ocular homeostasis. Oxidative stress has been proposed as one of the contributors to these processes, as cumulative oxidative damage may promote structural alterations in lens proteins and reduce tissue elasticity over time. Because presbyopia shares some biological features with other age-related ocular changes, including processes linked to lens ageing, interest has grown in interventions that may support the visual system through antioxidant and cytoprotective mechanisms [7,8].

Among nutritional candidates, berry-derived phytochemicals have attracted particular attention. Anthocyanin-rich preparations have been investigated for their effects on ocular and vascular function due to their antioxidant, anti-inflammatory, and microcirculatory properties [9,10,11]. However, the available evidence is heterogeneous and largely focuses on general visual fatigue, retinal function, and other ocular conditions rather than on presbyopia itself. Thus, despite the popularity of berry supplements for eye health, controlled clinical evidence supporting their use in the treatment of presbyopia remains limited [12,13].

In addition to anthocyanins, iridoids may be relevant in this context. Iridoid-containing fruits have been reported to exhibit antioxidant and anti-inflammatory activity, suggesting that they may complement the effects of polyphenol-rich berry extracts [14,15,16,17,18,19]. This is relevant because presbyopia is unlikely to depend on a single molecular pathway; rather, it reflects long-term, age-related disturbances in tissue structure and homeostasis. Therefore, combining phytochemicals with partly distinct biological profiles may offer a broader nutritional strategy than using a single extract alone.

This rationale is also supported by our previous studies showing that loganic acid, an iridoid-related constituent of cornelian cherry (*Cornus mas* L.), reduced intraocular pressure after oral administration in a rabbit model [20].

The formulation investigated in this study was a double-standardised mixture of extracts from *Aronia melanocarpa*, *Vaccinium myrtillus*, and *Lonicera caerulea* (AKB), containing anthocyanins at not less than 25% and iridoids at not less than 4.5%. This combination was selected to provide a complementary phytochemical profile: *Aronia* and *Vaccinium* are recognised as rich sources of anthocyanins and other phenolic compounds, whereas *Lonicera caerulea* contributes iridoids that are less typical of conventional berry-based eye-health supplements. Rather than assuming a confirmed synergistic mechanism, we considered this combined formulation as a biologically plausible, standardised nutritional intervention that could affect selected aspects of visual function and systemic processes potentially relevant to ocular ageing.

An additional challenge in this field is the scarcity of biomarkers that can bridge clinical outcomes to underlying biological processes in human presbyopia research. In the present study, we included exploratory circulating markers previously considered potentially relevant to lens biology and ocular homeostasis, including αA-crystallin, αB-crystallin, aldehyde dehydrogenase 1A1 (ALDH1A1), and TRPV4, together with selected oxidative stress- and glycation-related parameters. Importantly, these biomarkers were assessed as indirect and exploratory measures, rather than as proof of a specific mechanism operating in the crystalline lens.

Therefore, the scientific gap addressed in this study was twofold: first, the lack of controlled clinical trials evaluating standardised oral berry-based formulations in presbyopia; and second, the limited integration of functional ophthalmic outcomes with exploratory systemic biomarker assessment in this setting. To address this gap, we conducted a randomised, double-blind, placebo-controlled, two-period crossover trial to determine whether AKB supplementation is associated with improvements in selected visual-function outcomes in adults with presbyopia, with exploratory assessment of circulating biomarkers potentially relevant to lens and ocular homeostasis.

## 2. Materials and Methods

### 2.1. Chemicals

Acetonitrile, methanol, and formic acid for HPLC were obtained from Merc (Darmstadt, Germany). The standards of phenolic compounds and iridoids were purchased from Extrasynthese (Genay, France).

### 2.2. Analysis of Compounds by HPLC-PDA Method

Quantification of compounds was performed by HPLC-PDA (Dionex system; Germering, Germany) as described previously [21,22]. The analysis was performed under the following conditions: separation on a Cadenza Imtakt column CD–C18 (75 × 4.6 mm, 5 μm) with a guard column at 30 °C; sample injection volume, 20 μL; elution time, 30 min; flow rate, 1 mL/min. The mobile phase consisted of aqueous 4.5% formic acid (A) and 100% acetonitrile (B). Elution was as follows: 0–1 min 5% B in A, 1–20 min 25% B in A, 20–21 min 100% B, 21–26 min 100% B, 26–30 min 5% B in A. The compounds were detected at 245 nm (iridoids), 320 nm (phenolic acids), 360 nm (flavonols), and 520 nm (anthocyanins) and quantified using linear regression equations based on external standards. The results were expressed as g per 100 g dry weight (dw).

### 2.3. Study Group Description and Inclusion Criteria

The study involved thirty volunteers.

Participants were eligible if they were over 50 years of age and had presbyopia, best corrected visual acuity of at least 20/40, spherical refraction between −3.0 and +3.0 diopters, and a cylinder correction not exceeding ±3.0 diopters. Exclusion criteria included a history of ocular surgery within the 12 months preceding enrollment, age below 50 years, or the presence of ocular diseases such as macular degeneration, diabetic retinopathy, retinal vein occlusion, glaucoma, or other significant acquired or hereditary eye conditions. Individuals with neurological disorders affecting visual fields were also excluded. Eyes with a spherical equivalent lower than −6.0 D or higher than +3.0 D, as well as those requiring cylinder correction beyond ±3.0 D, did not qualify. Additional exclusion criteria covered patients with hypotension, severe circulatory failure, vascular endothelial disorders that might influence optic nerve head perfusion, as well as gastrointestinal conditions such as hyperacidity or peptic ulcer disease.

During the initial medical screening, three candidates were excluded based on these criteria. After the diagnostic procedures had started, but before the administration of study products, two participants were diagnosed with early glaucoma, and another was found to have vision impairment secondary to a systemic disease. In addition, one person withdrew after the first phase of the study.

Ultimately, 23 participants completed the full study protocol, allocated into two subgroups of 10 and 13 individuals.

### 2.4. Devices Used for the Study

Intraocular pressure was measured using the applanation method with a slit lamp after anaesthetising the cornea with Alcaine™ drops (0.5% proparacaine solution, Alcon Laboratories, Inc., Fort Worth, TX, USA).

Contrast testing was performed on Pelli-Robson charts (Gima S.p.A., Gessate, Italy) at 3 m and 40 cm.

The visual field test was performed on a Zeiss Humphrey Field Analyzer model 740i (Carl Zeiss Meditec, Inc., Dublin, CA, USA) using the 24-2 SITA fast programme.

Visual evoked potentials (VEP) were tested using a Tomey EP-1000 device, (Tomey Corporation, Nagoya, Japan) Version 3.2.0.

Angio-OCT was performed using the SD-OCT RTVUE XR AVANTI (Optovue, Inc., Fremont, CA, USA).

Blood tests were performed using an Agilent BioTek 50TS microplate washer and an Agilent BioTek Synergy H1 multimode microplate reader (Agilent Technologies, Inc., Santa Clara, CA, USA).

### 2.5. Description of the Study

The study protocol was approved by the Local Bioethics Committee of the Medical University of Wrocław (approval Nos. KB-672/2022, 8 September 2022, and KB-258/2025, 15 July 2025). All procedures were conducted in accordance with the principles of Good Clinical Practice (GCP) and the Declaration of Helsinki. Written informed consent was obtained from all participants. The study was retrospectively registered at ClinicalTrials.gov (Identifier: NCT07382882, 3 November 2022) to enhance transparency; prospective registration was not required under Polish regulations for this dietary-supplement intervention at the time the study commenced.

The trial was a randomised, double-blind, crossover study conducted in two phases separated by a washout period. In both phases (Phase 1 and Phase 2), the same ophthalmological and biochemical parameters were assessed before the start and after the completion of supplementation with the assigned preparation. Results from both phases were subsequently analysed. This study was designed as an exploratory proof-of-concept crossover trial. No formal a priori sample size calculation was performed because reliable effect-size estimates for this combined berry formulation in presbyopia were not available at the time of study design. Therefore, the results should be interpreted as preliminary and hypothesis-generating.

Phase 1 lasted 6 weeks (42 days), after which participants underwent a 5-week washout period. Phase 2 followed the same scheme and lasted 6 weeks (42 days). Throughout each phase, participants ingested one capsule of either the test product (AKB) or a placebo twice daily. Each AKB capsule contained a standardised extract of aronia, honeysuckle berry, and blackcurrant in a 2:1:1 ratio. Placebo capsules, also taken twice daily, consisted of maltodextrin with natural caramel colouring.

A crossover design enables the direct comparison of two interventions—here, the active preparation and placebo—within the same group of participants. Each subject receives both treatments in random order at different times, which allows evaluation of efficacy and safety while minimising the impact of inter-individual variability.

Ophthalmological assessments were performed prior to initiating supplementation and again in the final week of each treatment phase. The examinations included measurement of intraocular pressure, contrast sensitivity at near and distance, visual field testing, visual evoked potentials (VEP), and angio-OCT (optical coherence tomography with angiographic mode). In addition, blood samples were collected for biochemical analysis.

### 2.6. Blood Tests

The collected blood was prepared for further testing as follows: collected in serum storage tubes, left to stand for 30–60 min at room temperature, then centrifuged at 4000 rpm for 5 min in a centrifuge, then the serum was pipetted into small tubes and placed in a freezer. room temperature, then centrifuged at 4000 rpm for 5 min in a centrifuge, then the serum was pipetted into small tubes and placed in a freezer at −80 degrees.

Systemic biomarkers were selected as non-invasive exploratory measures potentially related to oxidative stress, glycation, protein homeostasis, and pathways of possible relevance to lens and ocular ageing. Because direct sampling of ocular tissues or fluids is not feasible in this type of clinical study, serum-based assessment was used as a pragmatic approach for hypothesis generation rather than as a direct readout of intraocular mechanisms.

The following tests were used to examine the collected blood: Superoxide Dismutase Assay Kit, Catalase Assay Kit, Glutathione Peroxidase Assay Kit Cayman Chemical, Ann Arbor, MI, USA. Connexin-43 EH2910 Human CX43 (Connexin 43) ELISA Kit, Advanced glycation end products (AGEs), Carboxymethyl Lysine (CML), Alpha A Crystallin, Alpha B Crystallin, Human ALDH1A1 (Retinal dehydrogenase 1) ELISA Kit (Fine Test, Wuhan, China); Human Transient receptor potential cation channel subfamily V member 1 (TRPV1) ELISA Kit, Carboxyethyl Lysine (CEL) (Sunlong Biotech Co., Ltd., Hangzhou, China. Human Transient Receptor Potential Cation Channel Subfamily V Member 4 (TrpV4) ELISA Kit Cusabio Technology LLC, Houston, TX, USA). Blood samples from 22 patients were tested.

### 2.7. Immunochemical Determination of Biochemical Analytes Concentration

CRYAA, CRYABB, Cx-43, ALDH1 and CML serum concentrations were determined by the enzyme-linked immunosorbent technique (ELISA) using commercially available kits (FineTest^®^ ELISA Kits, Wuhan Fine Biotech Co., Ltd., Wuhan, China; Cat. No.: EH1621 (CRYAA), EH0759 (CRYAB), EH2910 (Cx-43), EH1002 (ALDH1) and EH4116 (CML)). The Sunlong Biotech Co., Ltd., Hangzhou, China) kits were used to determine serum concentrations of TRPV1 (Cat. No.: SL3380Hu) and CEL (Cat. No.: SL2660Hu). TRPV4 levels were measured using a Cusabio (Wuhan Huamei Biotech Co., Ltd., Wuhan, China) ELISA kit (Cat. No.: CSB-E14199h). Absorbance readings were created using the Agilent BioTek Synergy H1 multimode microplate reader at a wavelength of 450 nm.

### 2.8. Determination of Oxidative Stress Parameters

SOD activity in serum samples was measured using a Superoxide Dismutase Assay Kit (Item no. 706002, Cayman Chemicals, Ann Arbor, MI, USA), which uses a tetrazolium salt to detect superoxide radicals. It is produced by xanthine oxidase and hypoxanthine. Collected samples were diluted 5-fold and analysed following the assay protocol. Absorbance was measured at 450 nm (Agilent BioTek Synergy H1 multimode microplate reader). Units of SOD activity were calculated from a standard curve using purified bovine erythrocyte SOD enzyme. The activity of SOD in plasma was expressed as U/mL. One unit of SOD activity is defined as the amount of enzyme needed to dismutase 50% of available superoxide radicals.

CAT activity was determined with Catalase Assay Kit (Item no. 707002, Cayman Chemicals, Ann Arbor, MI, USA) which is based on the reaction of the enzyme with methanol in the presence of H_2_O_2_ during which formaldehyde is produced and measured colorimetrically at 540 nm (BioTek Cytation 5 Cell Imaging Multimode Reader, BioTek Instruments Inc., Winooski, VT, USA) with 4-amino-3-hydrazino-5-mercapto-1,2,4-triazole as the chromogen. ACT activity was expressed as nmol/min/mL (one unit is defined as the amount of enzyme that will cause the formation of 1.0 nmol of formaldehyde per minute at 25 °C).

GPx activity was measured with the Glutathione Peroxidase Assay Kit (Item no. 703102, Cayman Chemicals, Ann Arbor, MI, USA). The test measures the decrease in absorbance at 340 nm during the oxidation of NADPH to NADP+, which is formed upon reduction of oxidised glutathione by glutathione reductase. The rate of decrease in absorbance at 340 nm is directly proportional to the GPx activity in the sample. The activity of GPx was expressed as nmol/min/mL (one unit is defined as the amount of enzyme that will cause the oxidation of 0.1 nmol of NADPH to NADP+ per minute at 25 °C).

### 2.9. Statistical Methods

To analyse the effect of the administered preparation on the eye’s physiological parameters, measures of central tendency (arithmetic mean, median, and mode) and measures of dispersion (standard deviation, variance, and range) were used. A nonparametric test—the Wilcoxon test—was performed to compare the means of two measurements within the same group (dependent samples), with a probability of error (*p*-value) of 0.05. Pearson’s correlation coefficient (r) was used to verify the direction and strength of the relationship between individual variables. IBM SPSS Statistics software (version 29.0; IBM Corp., Armonk, NY, USA) was used to analyse and process the data.

To minimise confounding and inter-individual variability, the study used a randomised, double-blind, placebo-controlled, two-period crossover design with a washout period, so that each participant served as their own control. Study procedures and assessment time points were standardised across both intervention periods.

Given the exploratory nature of the study, the modest sample size, and the crossover design, the statistical analysis was intentionally focused on prespecified within-subject and treatment-related comparisons rather than on highly parameterised multivariable models to avoid overfitting and unstable estimates.

### 2.10. Sample Size Considerations

This study was designed as an exploratory proof-of-concept crossover trial. A formal a priori sample size calculation was not performed because reliable estimates of the expected treatment effect and within-subject variability for this specific AKB formulation in presbyopia were not available at the time of study design.

Therefore, the present findings should be interpreted as preliminary and hypothesis-generating. The observed data may serve as pilot estimates for future formal sample size calculations in adequately powered confirmatory trials, which should be based on a prespecified primary endpoint and the within-subject variance appropriate for a crossover design.

## 3. Results

Chokeberry, honeysuckle berry, and bilberry are often referred to as superfruits due to their exceptionally high content of biocomponents, primarily polyphenols, with broad biological properties [23,24,25,26]. Honeysuckle berries also contain iridoids, including secoiridoids, which are not found in chokeberries or blueberries. The qualitative composition of polyphenolic and iridoid compounds in the AKB extract is comparable to that of fresh fruits, as reported in the literature. However, the concentration of these compounds in the AKB extract is many times greater than that in fresh fruit or fruit products. Therefore, AKB extract may be an attractive dietary supplement used in the treatment of many medical conditions.

### 3.1. Composition of the AKB Preparation

The AKB preparation is a mixture of extracts from *Aronia melanocarpa* (Michx.) Elliott (A), *Lonicera caerulea* L. (K), and *Vaccinium myrtillus* L. (B) in a ratio of 2:1:1. The details of the composition of the AKB fruit extracts were previously described by [22]. The AKB extract, double standardised for anthocyanin (not less than 25%) and iridoid (not less than 4.5%) content, has been registered as a dietary supplement with the Chief Sanitary Inspectorate (2022/08/09/SD/6228DF). The AKB dietary supplement was administered in a dose of two capsules per day, 400 mg in each capsule.

**Figure 1:**
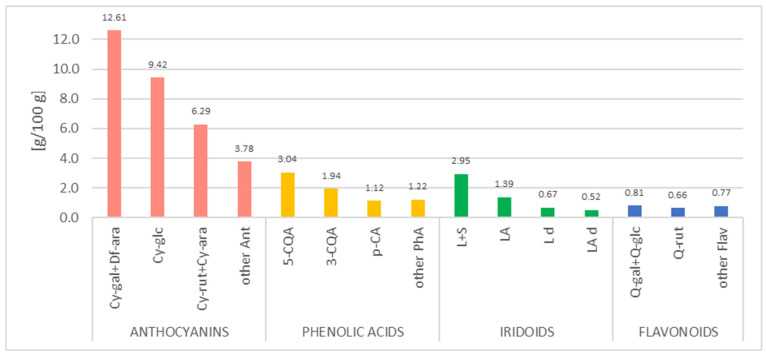
The content (g/100 g dry mass) of main phenolic and iridoid compounds of the AKB extract by HPLC-PDA. Abbreviations: Cy-gal+Df-ara—cyanidin 3-O-galactoside + delphinidin 3-O-arabinoside; Cy-glc—cyanidin 3-O-glucoside; Cy-rut+Cy-ara—cyanidin 3-O-rutinoside + cyanidin 3-O-arabinoside; Ant—anthocyanins; 5-CQA—5-caffeoylquinic acid; 3-CQA—3-caffeoylquinic acid; p-CA—p-coumaric acid isomer; PhA—phenolic acids; L+S—loganin + sweroside; LA—loganic acid; Ld—loganin derivative; LAd—loganic acid derivative; Q-gal+Q-glc—quercetin 3-O-galctoside + quercetin 3-O-glucoside; Q-rut—quercetin 3-O-rutinoside; Flav—flavonols.

Figure 1 and Figures S1–S3 and Table S1 show the composition of the AKB extract after HPLC analysis. Three groups of phenolic compounds: anthocyanins, hydroxycinnamic acids, and flavonols and iridoids were quantified. Chemical structures of the main compounds found in AKB preparation are shown in Supplementary Materials Figure S4.

### 3.2. Intraocular Pressure Test Results

All patients had normal intraocular pressure before administration of AKB. This condition was maintained after administration of the drug. A slight increase in pressure was also observed after administration of the preparation (in both the left and right eyes) compared with the initial measurement. The most common intraocular pressure value in the examination after 6 weeks of taking the preparation was 12 mm Hg. On average, there was an increase in intraocular pressure in both the right and left eyes after taking the preparation. In the left eye, the increase averaged 0.26 mm Hg, and in the right eye, 0.13 mm Hg.

**Figure 2:**
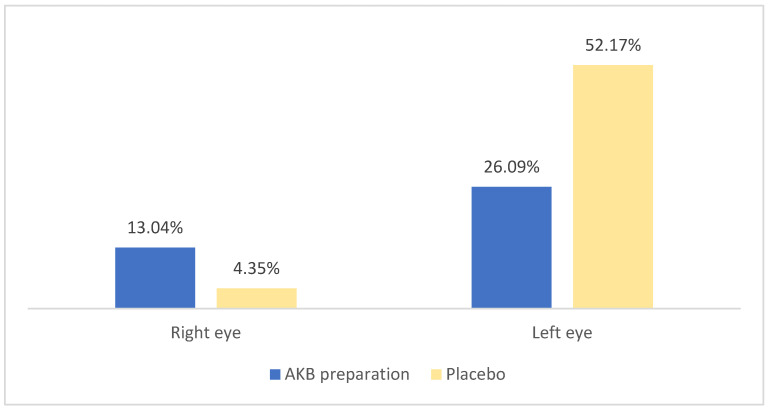
Average increase in intraocular pressure after taking AKB and a placebo.

Before the study, all patients had normal intraocular pressure before receiving the placebo. The most common intraocular pressure value in the second test was 14 mm Hg. For the left eye, this value was 13 mm Hg. On average, both the right and left eyes showed an increase in intraocular pressure after taking the placebo. In the left eye, the increase averaged 0.52 mm Hg, and in the right eye, 0.04 mm Hg. The results of the intraocular pressure examination are shown in Figure 2.

### 3.3. Contrast Sensitivity Test Results

Improvement after taking AKB was reported by 57% of patients who previously had problems with contrast sensitivity in the left eye. The average improvement in the left eye was 24% across the entire study group, regardless of whether patients had contrast sensitivity issues before the study. No significant improvement was observed in the right eye in patients with pre-study contrast sensitivity problems.

### 3.4. Near Contrast Test Results

People who had problems with near contrast sensitivity at baseline (before the study) were included in the analysis. Their results were analysed.

**Figure 3:**
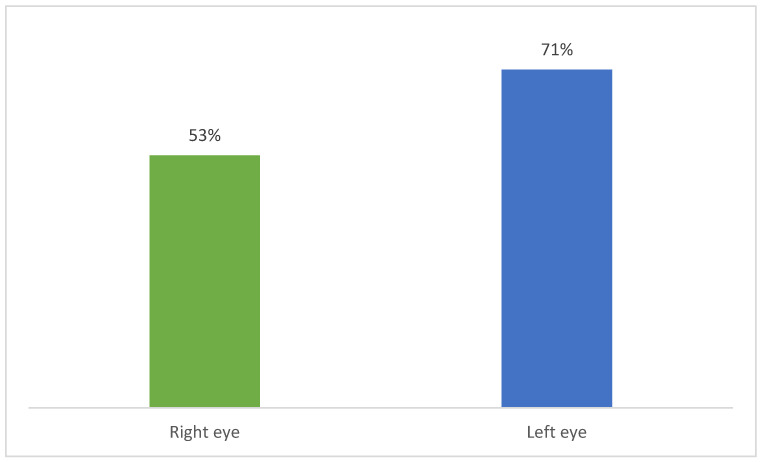
Improvement after taking AKB—contrast sensitivity.

An improvement was observed after AKB treatment in the left eye in 71% of patients (10 people) with contrast sensitivity problems in near vision. The average improvement in the left eye was 26%. Improvement was also observed after AKB treatment in the right eye in 53% of patients (8 people) with near contrast sensitivity problems. The average improvement in the right eye was 20%. The improvement in contrast sensitivity after taking AKB is shown in Figure 3.

The following changes were observed in the placebo control group:

Improvement in OL after placebo administration was observed in 64% of patients with near contrast sensitivity problems. The average improvement in OL was 15%.

Improvement in OP after placebo administration was observed in 53% of patients with near-vision contrast sensitivity problems. The average improvement in OP was 22%.

**Figure 4:**
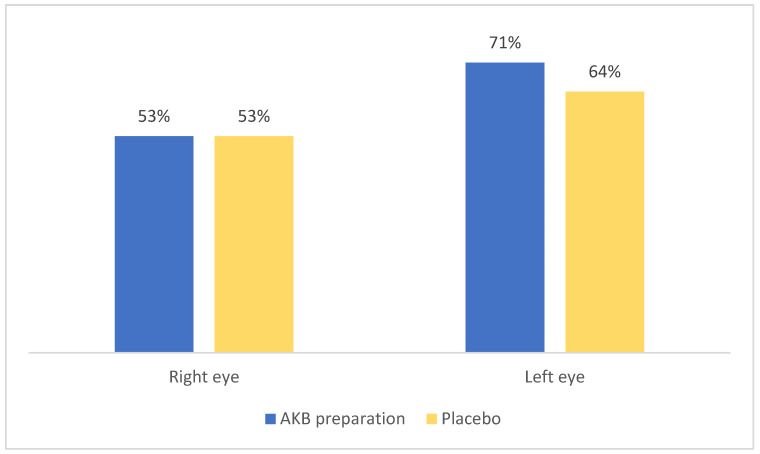
Improvement after taking AKB vs. placebo control group—near contrast sensitivity.

The improvement after taking AKB vs. placebo in the control group—near contrast sensitivity—is shown in Figure 4.

Improvement after taking AKB was reported in 80% of patients who previously had problems with contrast sensitivity at near in either eye. Improvement after taking a placebo was reported in 71% of patients who previously had problems with contrast sensitivity at near in either eye.

### 3.5. Results of the Visual Field Test

The test allows for the determination of defects in the visual field. The determination of smaller defects allows the colour scale to be presented as numbers—decibels.

In our study, we assessed MD (dB) (mean deviation) and PSD (dB) (pattern standard deviation) values.

Changes in MD values were analysed in relation to baseline values. An increase in MD values was interpreted as favourable, indicating improved retinal sensitivity and reduced visual field defects.

The PSD (Pattern Standard Deviation) parameter measures the irregularity of defects in the field of vision. The lower the value (≤2 dB), the more physiological the image of the field of vision. An increase in PSD may suggest the presence of pathological defects (e.g., in glaucoma—PSD is used to determine progression in the intermediate stage). The pattern standard deviation (PSD) is a measure of focal defects. It is determined by comparing the differences between adjacent points. Higher values indicate more focal losses, while lower values may indicate no losses or scattered losses.

The differences observed between the study and placebo groups may indicate the potential efficacy of AKB in improving retinal sensitivity and reducing visual field defects (neuroprotective or functional effects: improved retinal sensitivity or nerve conduction).

Visual field assessment was performed using the Humphrey Field Analyzer to determine the Mean Deviation (MD) and Pattern Standard Deviation (PSD) parameters. The MD parameter, reflecting overall retinal sensitivity, increased by 19.9% in the right eye and 6.3% in the left eye after AKB supplementation, whereas in the placebo group, a decrease of 1.3% in the right eye and a modest increase of 3.2% in the left eye were observed. These results suggest a numerical difference in MD changes between AKB and placebo, particularly in the right eye; however, given the modest magnitude of change, inter-eye variability, and exploratory study design, this finding should be interpreted cautiously.

The PSD parameter, which evaluates the regularity of focal visual field defects, remained within the normal range (≤2 dB) in the majority of cases. After AKB supplementation, 86.96% of right eyes and 82.61% of left eyes met the normal PSD criteria, compared with 91.30% and 82.61% in the placebo group, respectively. The differences between groups were small and statistically insignificant, indicating that AKB did not significantly influence PSD values. Overall, the results suggest a possible favourable change in MD after AKB supplementation, whereas PSD remained largely unchanged; the clinical relevance of these findings remains uncertain.

### 3.6. VEP Test Results

Assessment of parameters in the VEP test after administration of AKB:

Visual evoked potentials (VEP) were analysed to evaluate the conduction velocity and cortical response amplitude following AKB or placebo administration. After supplementation with the AKB preparation, a reduction in latency (L) was observed for both pattern sizes—by 1.06 ms (−0.9%) for Patt 1.0 and by 1.24 ms (−1.1%) for Patt 0.3—indicating faster transmission of visual signals within the visual pathway. Concurrently, the amplitude (A) increased by 0.55 µV (+5%) for Patt 1.0, suggesting enhanced cortical responsiveness, while a minor decrease (−0.89 µV; −1.9%) was recorded for Patt 0.3.

In contrast, placebo administration resulted in only minimal latency reduction (−0.035 ms for Patt 1.0 and −0.39 ms for Patt 0.3; −0.1% and −0.5%, respectively) accompanied by a marked decrease in amplitude (−0.995 µV; −17.3% for Patt 1.0 and −0.86 µV; −16.3% for Patt 0.3).

Overall, AKB supplementation was associated with directionally favourable changes in selected VEP parameters relative to placebo. However, these changes were modest, not uniform across pattern sizes, and should be interpreted as exploratory rather than as evidence of improved neural conduction or neuroprotection.

### 3.7. AngioOCT Results

The results of the GCC examination, retinal thickness, superficial and deep vascular density in aOCT are described in Table 1. In the group that received AKB, retinal ganglion cell thickness (GCC, whole image) increased by 0.94% in both eyes; ETDRS Grid increased by 0.94% in the right eye and by 0.61% in the left eye.

In the placebo group, the thickness of the retinal ganglion cell layer (GCC) (whole image) decreased by 0.0035% in the right eye and increased by 0.38% in the left eye; ETDRS Grid decreased by 0.4% in the right eye and by 0.07% in the left eye.

AKB supplementation was associated with a directionally favourable change in GCC thickness; however, given the exploratory design and modest sample size, this finding should be interpreted cautiously and cannot be taken as direct evidence of neuroprotection. A similar directional pattern was not observed in the placebo phase.

In the AKB group, retinal thickness (ETDRS Grid) decreased by 0.35% in the right eye and by 0.23% in the left eye. In the placebo group, retinal thickness (ETDRS Grid) decreased by 0.24% in the right eye and by 0.35% in the left eye.

There was no clear improvement in total retinal thickness. Minimal differences may be due to physiological variability or anti-oedema effects, but they are not clinically significant.

In the group that received AKB, the density of superficial vessels (whole image) decreased by 4.6% in the right eye and increased by 0.56% in the left eye; ETDRS Grid decreased by 5.48% in the right eye and by 5.67% in the left eye. In the placebo group, the density of superficial vessels (whole image) increased by 0.34% in the right eye and by 0.6% in the left eye; ETDRS Grid increased by 0.05% in the right eye and decreased by 1.87% in the left eye.

AKB was associated with a decrease in superficial vascular density in some measurements; the biological meaning of this finding is unclear and may reflect physiological variability, measurement noise, or a treatment-related effect requiring further study.

In the group that received AKB, deep vessel density (whole image) increased by 3.27% in the right eye and decreased by 0.65% in the left eye; ETDRS Grid increased by 1.9% in the right eye and decreased by 0.04% in the left eye.

In the placebo group, deep vessel density (whole image) increased by 0.22% in the right eye and by 0.15% in the left eye; ETDRS Grid increased by 0.81% in the right eye and decreased by 0.7% in the left eye.

AKB was associated with a numerical increase in deep vessel density in some measurements, particularly in the right eye, but this finding was not consistent across both eyes and should be interpreted cautiously.

Summarising the results of the effect on angioOCT: AKB was associated with directionally favourable changes in selected GCC and vascular OCT readouts, but these exploratory findings do not establish neuroprotection or a definite vascular benefit. However, the decrease in superficial vascular density may raise concerns and requires further observation—e.g., whether it is a regulatory, transient, or undesirable effect.

### 3.8. Blood Test Results

The blood test results are described in Table 2.

### 3.9. Biochemical Parameters After AKB Supplementation

The biochemical analyses were performed to verify whether the intake of the anthocyanin- and iridoid-standardised AKB extract produced measurable systemic effects on antioxidant defence, lens-related proteins, transient receptor potential (TRPV) channels, and advanced glycation end-products (AGEs). All parameters were assessed twice, before (Test 1) and after (Test 2) supplementation, under a crossover design versus placebo.

#### 3.9.1. Antioxidant Enzymes and Connexin-43

Serum SOD, CAT, and GPx activities showed no meaningful changes after AKB compared with placebo (Table 2), suggesting that the visual benefits were not driven by a measurable shift in systemic redox enzyme activity in this cohort. Connexin-43 (Cx-43) also remained stable, consistent with preserved systemic gap-junction–related homeostatic signalling over the intervention period.

#### 3.9.2. Lens Crystallins (CRYAA, CRYAB)

AKB supplementation was associated with lower circulating CRYAA and CRYAB (Table 2). Because elevated serum α-crystallins have been linked to cellular stress and/or lens protein leakage, this direction of change is compatible with improved lens protein homeostasis and aligns with the concurrent functional improvements (contrast sensitivity) and neuro-retinal readouts (VEP/OCT).

#### 3.9.3. Transient Receptor Potential Channels (TRPV-1 and TRPV-4)

TRPV1 showed a small decrease, whereas TRPV4 increased after AKB (Table 2). Given TRPV4’s role in lens volume/osmotic regulation and transparency maintenance, the observed increase in TRPV4 may represent a biologically plausible signal relevant to lens homeostasis; however, no direct functional mechanistic validation was performed in the present study. Inter-individual variability suggests that responsiveness may depend on baseline metabolic or genetic factors and warrants assessment in larger cohorts.

#### 3.9.4. Advanced Glycation and Oxidative-Modification Products

Systemic AGEs, CEL, and CML were not significantly altered by AKB (Table 2), indicating no detectable short-term effect on circulating glycoxidation markers. This does not exclude local ocular effects, but it suggests that the clinical improvements occurred without a measurable change in systemic glycation burden over six weeks.

#### 3.9.5. Integrative Interpretation

Taken together, the biochemical profile points to a selective, lens-relevant signature (↓α-crystallins, ↑TRPV4) with stable systemic antioxidant enzymes and glycation markers. This pattern supports the interpretation that AKB may “fine-tune” pathways related to lens homeostasis and visual signalling rather than acting as a broad systemic antioxidant in circulation. Future studies should test longer supplementation periods and include more proximal ocular matrices (e.g., tear fluid) to better capture local biochemical changes.

## 4. Discussion

Berries are widely recognised as sources of polyphenols, particularly anthocyanins, with potential relevance to ocular tissues, although human studies often use heterogeneous preparations, making it difficult to attribute effects to a defined chemical profile [27,28,29,30,31]. In this context, AKB is of interest because it is a double-standardised combination of *Aronia melanocarpa*, *Lonicera caerulea*, and *Vaccinium myrtillus*, providing both anthocyanins and iridoids in a chemically defined formulation. The literature on these species supports the biological plausibility of antioxidant and anti-inflammatory activity, but in the present study, these data serve only as background rationale and not as direct evidence for a specific mechanism in presbyopia [32,33,34,35,36].

Consistent with this rationale, our randomised, double-blind crossover trial suggests that AKB may be associated with modest changes in selected functional readouts relevant to presbyopia, most notably near contrast sensitivity and visual-field performance, together with directionally favourable electrophysiological (VEP) and OCT/angioOCT findings. However, these effects were generally modest; some changes were also observed during the placebo phase, and the findings should therefore be interpreted cautiously. The accompanying biochemical pattern, including lower circulating α-crystallins and higher TRPV4, may be compatible with pathways relevant to lens and retinal homeostasis; however, because these markers were assessed in serum, this interpretation remains indirect and exploratory.

Current management of presbyopia remains dominated by optical correction, while pharmacological and nutritional approaches are still emerging [2,8,37,38,39,40]. Previous studies have suggested that both lens-targeted pharmacological interventions and selected nutritional formulations may influence near-vision-related outcomes, but the evidence remains limited and heterogeneous. Accordingly, the present results should be interpreted as exploratory and positioned within an early-stage field rather than as confirmation of an established therapeutic strategy.

Experimental studies suggest that TRPV1/TRPV4-related pathways may contribute to lens hydrostatic-pressure regulation and age-related lens biomechanics, making them of potential interest in presbyopia research [1,41,42]. However, in the present study, TRPV4 was measured only in serum, so its relevance to ocular physiology remains uncertain [41].

Visual field testing is an important component of functional ophthalmic assessment and may be altered in disorders affecting the retina or optic nerve [43]. In the present study, AKB was associated with a more favourable change in mean deviation (MD), particularly in the right eye, whereas changes in pattern standard deviation (PSD) were small and not clearly different from placebo. This pattern may suggest a directional effect on overall visual-field performance, but the magnitude of the change was modest, and its clinical relevance remains uncertain.

Because PSD remained largely unchanged, the visual-field findings should not be interpreted as evidence of a robust effect on focal field defects. Rather, they may indicate a limited or exploratory signal that requires confirmation in larger studies with prespecified endpoints.

Previous studies with compounds such as citicoline have reported favourable effects on visual pathway function in other ophthalmic settings, for example, glaucoma [44,45,46]. However, such comparisons should be made cautiously, as the populations, interventions, and mechanistic context differ from those of the present exploratory presbyopia study.

Increased latency and reduced amplitude of visual evoked potentials (VEP), commonly found in ocular hypertension or open-angle glaucoma, suggest slowed nerve conduction in the visual pathways [47,48]. Previous studies have reported VEP changes after selected interventions in other ophthalmic conditions, but such comparisons should be made cautiously because the populations, interventions, and mechanistic context differ from those of the present exploratory presbyopia study [49,50,51].

In our study, AKB was associated with a greater reduction in latency, particularly for Patt 1.0, than placebo. A more favourable amplitude pattern was also observed for Patt 1.0, whereas the findings for Patt 0.3 were less consistent. These results may suggest a directional effect on selected VEP parameters; however, given the modest magnitude of change, the inconsistency across pattern sizes, and the exploratory nature of the study, they should be interpreted cautiously. Therefore, the present findings should not be considered direct evidence of improved neural conduction, enhanced cortical responsiveness, or neuroprotection, but rather as preliminary electrophysiological observations that require confirmation in larger studies.

Oxidative stress has been implicated in lens ageing and cataract-related processes [52,53,54]. In our study, however, AKB was not associated with measurable changes in serum SOD, CAT, or GPx activity, suggesting that the observed functional findings were not accompanied by a detectable short-term shift in systemic antioxidant enzyme status. Because these markers were measured in serum rather than ocular tissue, no direct conclusions about intraocular redox processes can be drawn.

Connexin-43 has been implicated in lens redox homeostasis [54], but AKB did not materially affect circulating Cx-43 in the present study.

In the present study, AKB supplementation was associated with higher circulating TRPV4 levels. However, because TRPV4 was assessed in serum rather than in ocular tissue, the functional relevance of this finding for lens physiology or visual outcomes cannot be determined from the current data and should be regarded as exploratory.

Similarly, α-crystallins are central to lens transparency and protein quality control [55]. We observed lower circulating CRYAA and CRYAB after AKB supplementation; however, the interpretation of this finding remains uncertain. Because these proteins were measured in blood and not in the lens, the observed changes cannot be taken as direct evidence of improved lens homeostasis, reduced oxidative stress, or decreased chaperone demand. Rather, they should be considered indirect and hypothesis-generating findings that warrant further study in more proximal ocular matrices.

AGEs accumulate with age in ocular tissues and have been implicated in lens epithelial dysfunction and cataract-related processes [42,56]. In the present study, AKB did not materially affect circulating CML, CEL, or total AGEs over the intervention period. This suggests that any functional changes observed in the study were not accompanied by a measurable short-term shift in systemic glycation markers, although local ocular effects cannot be excluded.

ALDH1A1, also known as retinaldehyde dehydrogenase 1 (RALDH1), is a multifunctional enzyme implicated in aldehyde detoxification, oxidative-stress responses, and retinoid metabolism in ocular tissues [57,58,59,60,61]. In the present study, circulating ALDH1A1 levels changed after supplementation; however, the biological interpretation of this observation remains unclear. Because ALDH1A1 was assessed in serum rather than in ocular tissue, its relationship to lens, corneal, or choroidal physiology cannot be inferred from the present data. For this reason, the ALDH1A1 findings should be regarded as exploratory and should not be overinterpreted mechanistically.

**Figure 5:**
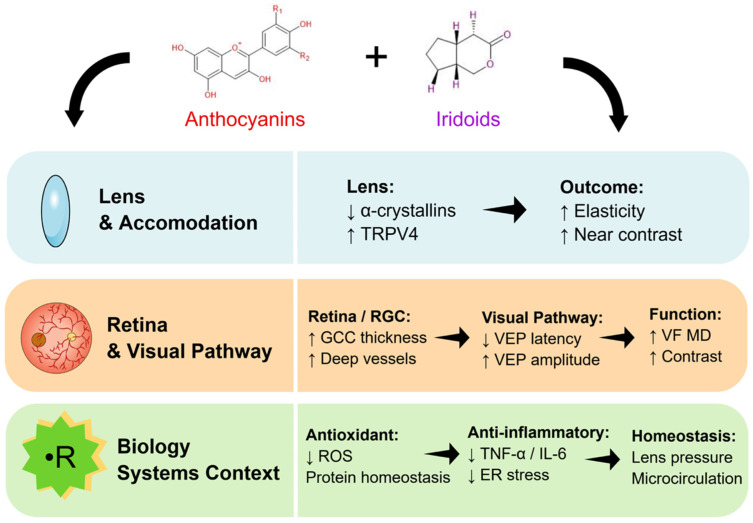
Hypothesized pathways potentially relevant to the observed findings, based on literature data and exploratory biomarker results.

More broadly, polyphenols, anthocyanins, and iridoids have been investigated in relation to several ophthalmic conditions and ocular stress pathways [19,20]; however, in the present study, these literature-based observations provide only biological context and do not directly explain the functional outcomes observed after AKB supplementation. A hypothesised literature-based framework of potentially relevant pathways is shown in Figure 5.

### Limitations and Future Perspectives

This study has several limitations. First, the sample size was small (n = 23), and no formal a priori power calculation was performed because reliable effect-size estimates for this specific AKB formulation in presbyopia were unavailable at the time of study design. Accordingly, the findings should be interpreted as preliminary and hypothesis-generating. Second, the intervention lasted only 6 weeks, which may be insufficient for a slowly progressive condition such as presbyopia. The study population was also restricted to adults aged 50 years or older, limiting the generalisability of the findings to younger individuals with earlier-stage presbyopia. Third, the evaluated biomarkers were measured in serum and should be regarded as indirect, exploratory indicators rather than direct measures of ocular processes. Fourth, although the crossover design reduced inter-individual variability, potential carryover effects cannot be fully excluded. In addition, multiple functional and biochemical outcomes were assessed in a modest sample, and future confirmatory studies should include prespecified primary endpoints and appropriate control for multiple comparisons.

The possibility of placebo-related effects, particularly in functional outcomes such as contrast sensitivity, cannot be fully excluded. Moreover, some of the observed changes were modest in magnitude and should therefore be interpreted cautiously. Differences between the right and left eyes may reflect biological variability, measurement noise, or limited statistical power rather than a consistent unilateral treatment effect. In addition, some functional outcomes showed changes in the same direction during the placebo phase, which further supports a cautious interpretation of any apparent treatment-related benefit.

Future studies should confirm these findings in larger, adequately powered trials with longer follow-up and broader patient populations. Mechanistic investigations should further examine pathways related to lens and retinal homeostasis, including TRPV4-associated signalling, and, where feasible, assess biomarkers in ocular fluids such as tear fluid or aqueous humour. Dose–response relationships, optimisation of AKB dosage, and potential interactions with other nutritional or therapeutic strategies for presbyopia also warrant investigation. Finally, future trials should include clinically meaningful outcomes, such as patient-reported visual function and quality of life.

## 5. Conclusions

This appears to be the first clinical study of a standardised combination of aronia, honeysuckle berry, and bilberry extracts containing both iridoid and polyphenolic fractions, evaluated in relation to VEP, VF, and OCT outcomes in adults with presbyopia. AKB supplementation was not associated with changes in intraocular pressure, but was associated with favourable changes in selected visual-function outcomes, including contrast sensitivity, MD in visual field testing, and selected VEP and angio-OCT parameters.

AKB supplementation was also associated with changes in circulating levels of CRYAA, CRYAB, ALDH1A1, and TRPV4. However, these findings should be interpreted cautiously, as the biomarkers were assessed in blood and serve only as indirect, exploratory correlates rather than direct measures of ocular processes. Thus, they do not establish a specific mechanism of action.

Overall, the present findings provide preliminary evidence that AKB may be associated with selected functional and systemic changes in adults with presbyopia. However, given the small sample size and the preliminary nature of the biomarker analyses, these findings should be interpreted cautiously and viewed as a basis for further research. Larger, adequately powered, and longer-term studies are needed to confirm the clinical relevance of these observations and to clarify their biological basis.

## Figures and Tables

**Table 1:** AngioOCT results. Abbreviations: GCC, ganglion cell complex; ETDRS, Early Treatment Diabetic Retinopathy Study grid; aOCT, angiographic optical coherence tomography.

GCC thickness	Right eye		Left eye	
	AKB	Placebo	AKB	Placebo
superior	0.51	0.03	1.33	1.14
inferior	0.70	−0.44	0.25	−0.57
whole image	0.94	−0.004	0.94	0.38
ETDRS Grid	0.94	−0.43	0.61	−0.07
Retinal thickness	Right eye		Left eye	
	AKB	Placebo	AKB	Placebo
fovea	−1.72	−0.94	−3.45	−2.51
para fovea	0.14	−0.11	−0.18	0.07
peri-fovea	No data	No data	No data	No data
ETDRS Grid	−0.35	−0.24	−0.23	−0.35
Superficial density	Right eye		Left eye	
	AKB	Placebo	AKB	Placebo
Superior	−5.50	−3.68	0.84	1.05
inferior	−7.98	−0.35	1.12	0.18
whole image	−4.60	0.34	0.56	0.60
ETDRS Grid	−5.48	0.05	−5.67	−1.87
Deep density	Right eye		Left eye	
	AKB	Placebo	AKB	Placebo
superior	3.06	7.35	−0.60	0.00
inferior	3.57	0.41	−0.63	0.15
whole image	3.27	0.22	−0.65	0.15
ETDRS Grid	1.90	0.81	−0.04	0.70

**Table 2:** Biochemical test results. Abbreviations: SOD, superoxide dismutase; CAT, catalase; GPx, glutathione peroxidase; Cx-43, connexin-43; CRYAA, alpha A-crystallin; CRYAB, alpha B-crystallin; TRPV1, transient receptor potential vanilloid 1; TRPV4, transient receptor potential vanilloid 4; AGEs, advanced glycation end products; CEL, Nε-carboxyethyllysine; CML, Nε-carboxymethyllysine; ALDH1A1, aldehyde dehydrogenase 1 family member A1.

	Test 1						Test 2					
	SOD	CAT		GPx		Cx-43	SOD	CAT		GPx		Cx-43
Average	2.041	14.197		44.075		2.835	1.987	13.05		43.43		2.545
Standard error	0.169	1.935		3.054		0.144	0.206	1.204		2.844		0.136
Median	1.834	10.860		44.826		2.871	1.919	12.23		41.76		2.471
Interquartile range	1.313	10.027		18.338		1.118	1.100	5.954		17.31		0.718
	Test 1						Test 2					
	CRYAA			CRYAB			CRYAA			CRYAB		
Average	13.378			3.037			12.597			2.857		
Standard error	0.706			0.143			0.775			0.152		
Median	12.596			2.939			12.036			2.833		
Interquartile range	5.546			1.074			3.533			1.185		
	Test 1						Test 2					
	TRPV-1			TRPV-4			TRPV-1			TRPV-4		
Average	840.906			68.976			798.866			74.579		
Standard error	55.218			9.615			35.951			15.940		
Median	788.842			63.428			795.025			56.304		
Interquartile range	159.018			59.726			95.757			63.642		
	Test 1						Test 2					
	AGEs		CEL		CML		AGEs		TARGET		CML	
Average	133.145		749.958		135.262		133.103		749.288		141.823	
Standard error	4.538		33.742		14.536		4.450		28.849		22.751	
Median	133.021		732.882		130.948		133.410		724.288		114.841	
Interquartile range	35.042		140.076		102.262		30.500		116.185		113.457	
	ALDH1A1											
	Test 1						Test 2					
Average	31.852						18.417					
Standard error	2.435						0.969					
Median	28.680						18.268					
Interquartile range	17.085						7.730					

## Data Availability

The original contributions presented in this study are included in the article/Supplementary Materials. Further inquiries can be directed to the corresponding author.

## Supplementary Materials

The following supporting information can be downloaded at: https://www.mdpi.com/article/10.3390/nu18061016/s1, Figure S1. The content (g/100 g dry mass) of other phenolic compounds (anthocyanins, phenolic acids, and flavonols) of the AKB extract was determined by HPLC-PDA. Figure S2. HPLC-PDA chromatogram (520 nm) of anthocyanins of the AKB extract. Figure S3. HPLC-PDA chromatograms (320 nm; 245 nm; 360 nm) of compounds (phenolic acids, iridoids, and flavonols) of the AKB extract. Figure S4. Chemical structures of the main compounds found in AKB preparation. Table S1. Anthocyanins, phenolic acids, iridoids, and flavonols content (mg/100 g fw) of the extract AKB by HPLC.

## Abbreviations

The following abbreviations are used in this manuscript:

AGEs	Advanced glycation end products
AKB	Mixture of standardised extracts from Aronia *melanocarpa* (chokeberry). *Lonicera caerulea* (honeysuckle/Kamchatka berry) and *Vaccinium myrtillus* (blueberry)
ALDH1A1 (RALDH1)	Retinaldehyde dehydrogenase 1
aOCT/angioOCT	Angiographic optical coherence tomography
CAT	Catalase
CEL	N6-Carboxyethyllysine
CML	N6-Carboxymethyllysine
CRYAA	Alpha A-crystallin
CRYAB	Alpha B-crystallin
Cx-43	Connexin-43
GPx	Glutathione peroxidase
GSH	Glutathione
LE	Left eye
LEC	Lens epithelial cells
MD	Mean deviation
PCO	Posterior capsule opacification
PSD	Pattern standard deviation
RA	Right eye
RAGE	Receptor for advanced glycation end-products
ROS	Reactive oxygen species
SOD	Superoxide dismutase
TRPV1	Transient receptor potential cation channel subfamily V member 1
TRPV4	Transient receptor potential cation channel subfamily V member 4
VEP	Visual evoked potentials
VF	Visual field
