# Supplementary material for: Standardized Berry Extract Improves Selected Visual Function Outcomes in Presbyopia: A Randomized, Double-Blind, Placebo-Controlled Crossover Trial with Exploratory Biomarker Analysis
Source: Nutrients. 2026 Mar 23;18(6):1016. doi: 10.3390/nu18061016 (PMC13028795; doi:10.3390/nu18061016)
Supplement: Supplementary file 1 [file nutrients-18-01016-s001.zip › Fig. S1_content of other phenolic compounds .pdf]

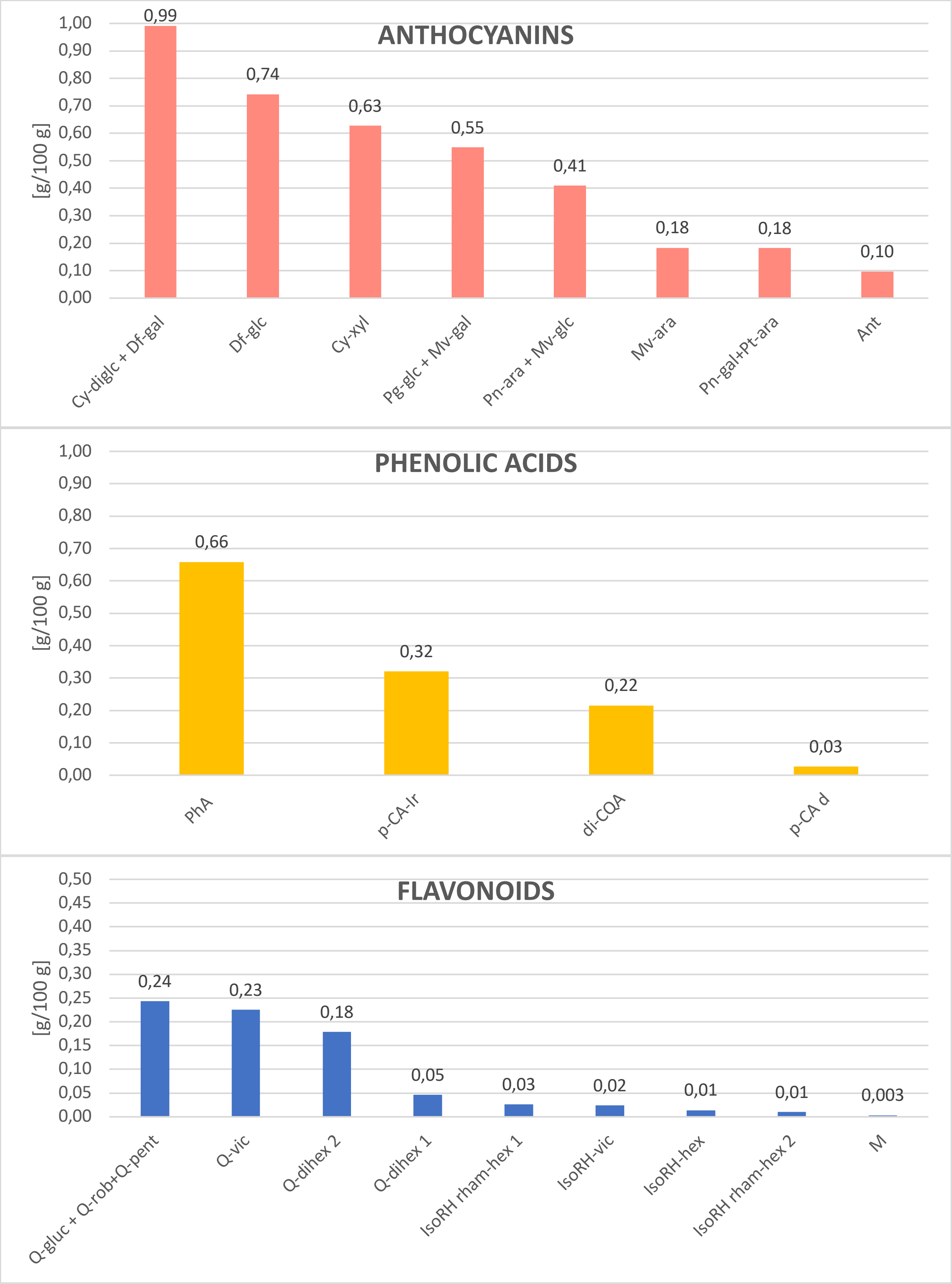

Figure S1. The content (g/100 g dry mass) of other phenolic compounds (anthocyanins, phenolic acids, and flavonols) of the AKB extract by HPLC-PDA. Abbreviations: **Cy-diglc + Df-gal**–cyanidin 3,5-*O*-diglucoside + delphinidin 3-*O*-galactoside, **Df-glc**–delphinidin 3-*O*-glucoside; **Cy-xyl**–cyanidin 3-*O*-xyloside; **Pg-glc + Mv-gal**–peonidin 3-*O*-glucoside + malvidin 3-*O*-galactoside; **Pn-ara + Mv-glc**–peonidin 3-*O*-arabinoside + malvidin 3-*O*-glucoside; **Mv-ara**–malvidin 3-*O*-arabinoside; **Pn-gal+Pt-ara**–peonidin 3-*O*-galactoside + petunidin 3-*O*-arabinoside; Ant–anthocyanins; **PhA**–other phenolic

acids; ***p*-CA-Ir**–*p*-coumaroyl iridoid glycosides; **di-CQA**–dicaffeoylquinic acid; ***p*-CA d**–*p*-coumaric acid isomer 2; **Q-gluc + Q-rob+Q-pent**–quercetin 3-*O*-glucuronide + quercetin 3-*O*-robinobioside + quercetin 3-*O*-pentoside; **Q-vic**–quercetin 3-*O*-vicianoside; **Q-dihex 2**–quercetin-dihexoside isomer 2; **Q-dihex 1**–quercetin-dihexoside isomer 1; **IsoRH rham-hex 1**–isorhamnetin rhamnosylhexoside isomer 1; **IsoRH-vic**–isorhamnetin 3-*O*-vicianoside; **IsoRH-hex**–Isorhamnetin pentosylhexoside; **IsoRH rham-hex 2**–Isorhamnetin rhamnosylhexoside isomer 2; **M**–Myricetin
