# Supplementary material for: Standardized Berry Extract Improves Selected Visual Function Outcomes in Presbyopia: A Randomized, Double-Blind, Placebo-Controlled Crossover Trial with Exploratory Biomarker Analysis
Source: Nutrients. 2026 Mar 23;18(6):1016. doi: 10.3390/nu18061016 (PMC13028795; doi:10.3390/nu18061016)
Supplement: Supplementary file 1 [file nutrients-18-01016-s001.zip › Fig. S2_chromatogram of anthocyanins.pdf]

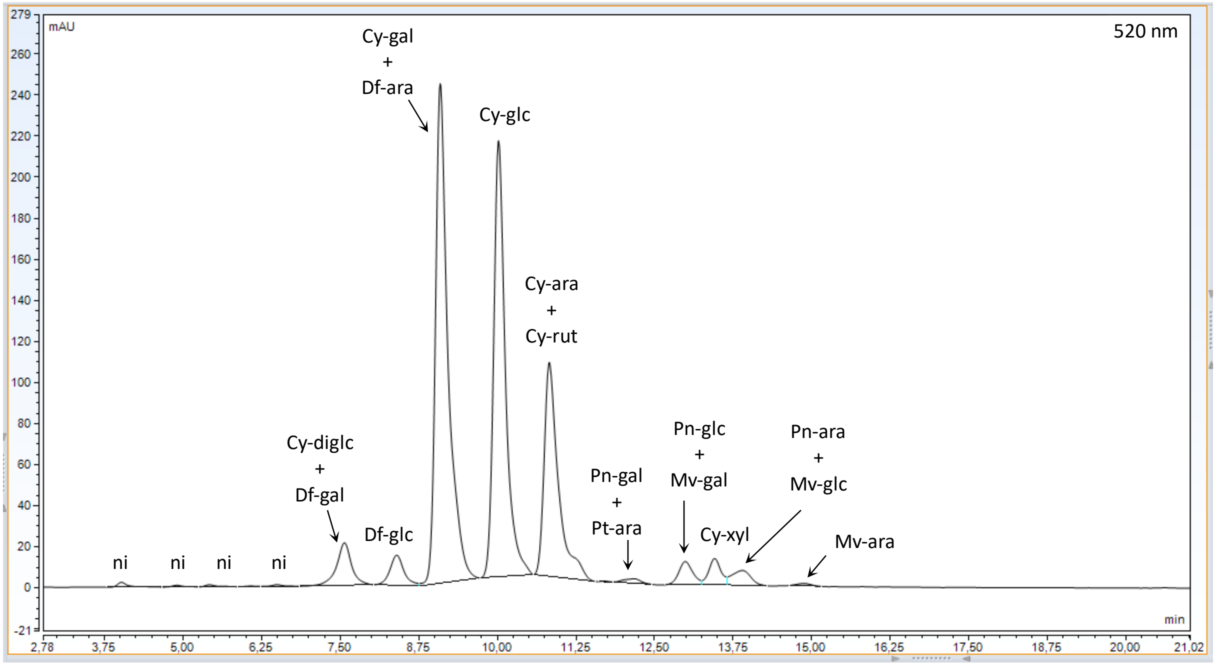

Figure S2. HPLC-PDA chromatogram (520 nm) of anthocyanins of the AKB extract. Abbreviations: **ni**–not identified; **Cy-diglc**–cyanidin 3,5-*O*- diglucoside; **Df-gal**–delphinidin 3-*O*-galactoside; **Df-glc**–delphinidin 3-*O*-glucoside; **Cy-gal**–cyanidin 3-*O*-galactoside; **Df-ara**–delphinidin 3-*O*-arabinoside; **Cy-glc**–cyanidin 3-*O*-glucoside; **Cy-ara**–cyanidin 3-*O*-arabinoside; **Cy-rut**–cyanidin 3-*O*-rutinoside; **Pn-gal**–peonidin 3-*O*-galactoside; **Pt-ara**– petunidin 3-*O*-arabinoside; **Pn-glc**–peonidin 3-*O*-glucoside; **Mv-gal**–malvidin 3-*O*-galactoside; **Cy-xyl**–cyanidin 3-*O*-xyloside; **Pn-ara**–peonidin 3-*O*-arabinoside; **Mv-glc**–malvidin 3-*O*-glucoside; **Mv-ara**–malvidin 3-*O*-arabinoside.
