# Supplementary material for: Standardized Berry Extract Improves Selected Visual Function Outcomes in Presbyopia: A Randomized, Double-Blind, Placebo-Controlled Crossover Trial with Exploratory Biomarker Analysis
Source: Nutrients. 2026 Mar 23;18(6):1016. doi: 10.3390/nu18061016 (PMC13028795; doi:10.3390/nu18061016)
Supplement: Supplementary file 1 [file nutrients-18-01016-s001.zip › Fig. S3_chromatograms of phenolic acids, iridoids, and flavonols.pdf]

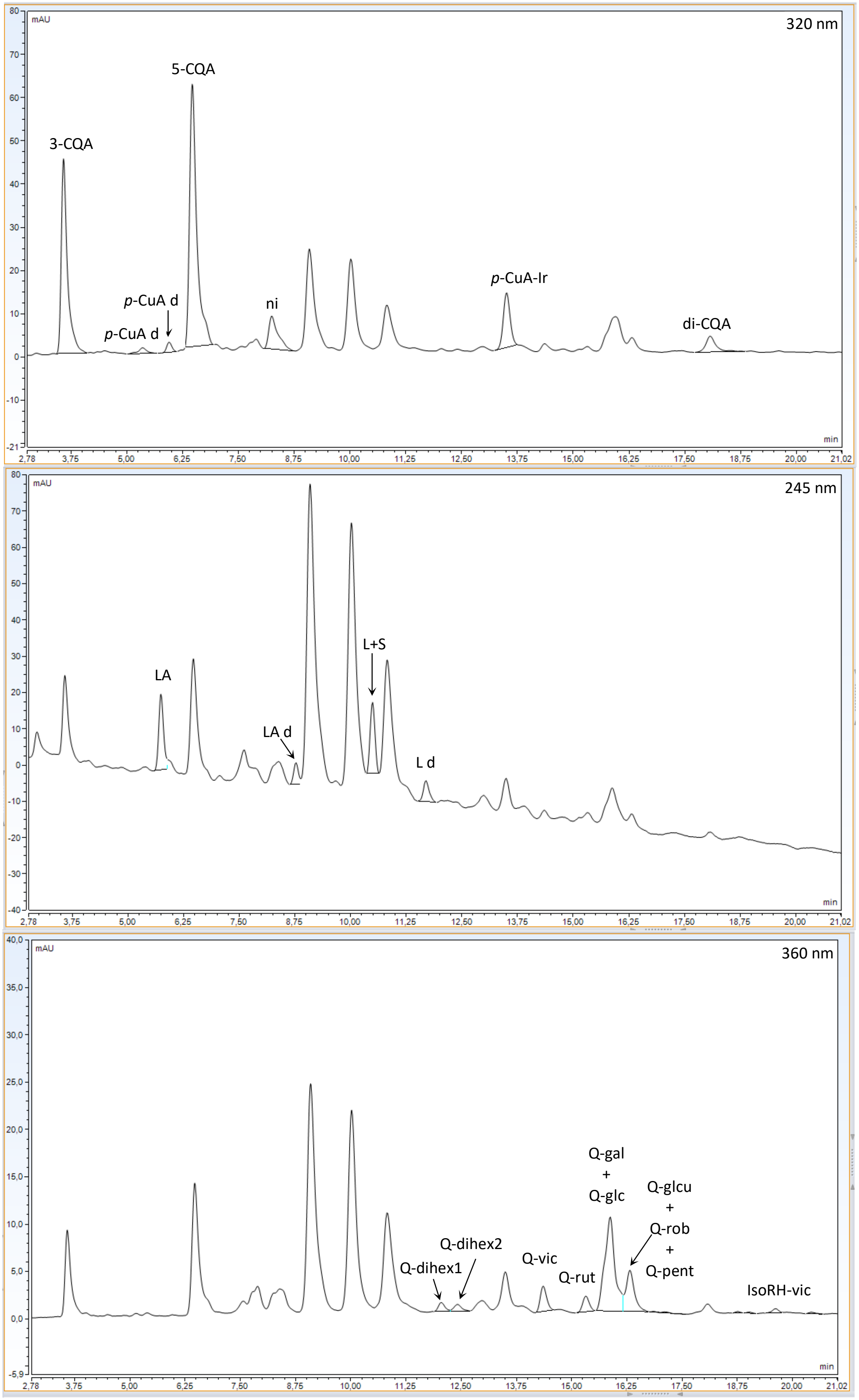

Figure S3. HPLC-PDA chromatograms (320 nm; 245 nm; 360 nm) of compounds (phenolic acids, iridoids, and flavonols) of the AKB extract. Abbreviations: **3-CQA**–3-caffeoylquinic acid; ***p*-CuA d**–p-coumaric acid derivative (1 and 2); **5-CQA**–5-caffeoylquinic acid; **ni**–not identified; ***p*-CuIr-g**–*p*-coumaroyl iridoid glycosides; **di-CQA**–dicaffeoylquinic acid; **LA**–loganic acid; **LA d**–loganic acid derivative; **L**–loganin: **S**–sweroside; **L d**–loganin derivative; **Q-dihex1**–quercetin-dihexoside isomer 1; **Q-dihex2**–quercetin-dihexoside isomer 2; **Q-vic**–quercetin 3-*O*-vicianoside; **Q-rut**–quercetin 3-*O*-rutinoside; **Q-gal**–quercetin 3-*O*-galctoside; **Q-glc**–quercetin 3-*O*-glucoside; **Q-glcu**–quercetin 3-*O*-glucuronide; **Q-rob**–quercetin 3-*O*-robinobioside; **Q-pent**–quercetin 3-*O*-pentoside; **30**–isorhamnetin 3-O-vicianoside;
