# Supplementary material for: Standardized Berry Extract Improves Selected Visual Function Outcomes in Presbyopia: A Randomized, Double-Blind, Placebo-Controlled Crossover Trial with Exploratory Biomarker Analysis
Source: Nutrients. 2026 Mar 23;18(6):1016. doi: 10.3390/nu18061016 (PMC13028795; doi:10.3390/nu18061016)
Supplement: Supplementary file 1 [file nutrients-18-01016-s001.zip › Fig. S4_chemical structures_AKB.pdf]

SUPPLEMENTARY MATERIALS

Chemical structures of main compounds found in AKB preparation

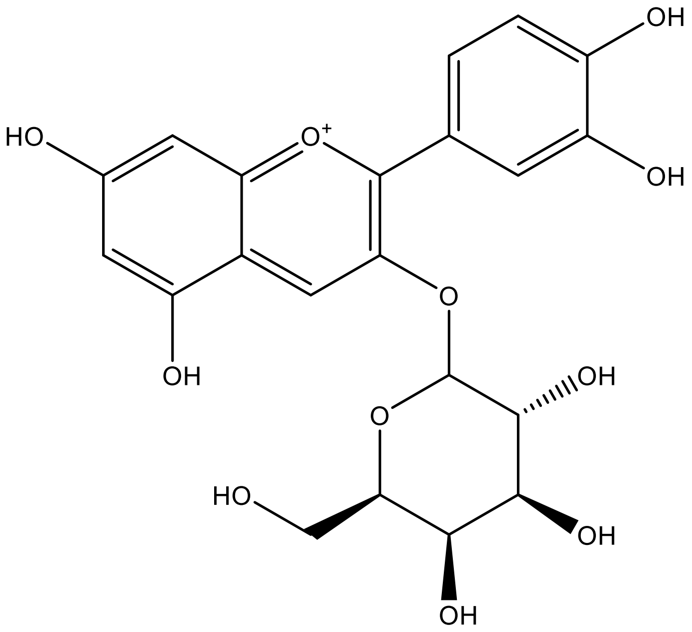

Cyanidin-3-O-galactoside

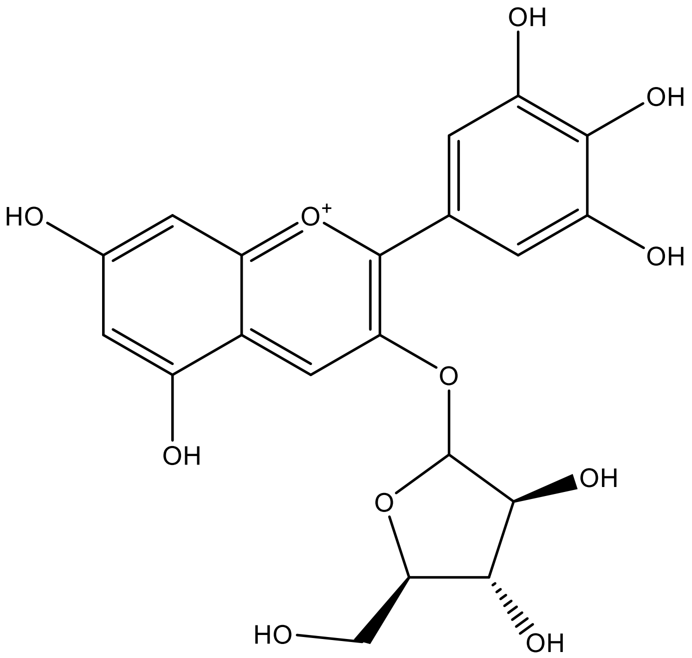

Delphinidin 3-O-arabinoside

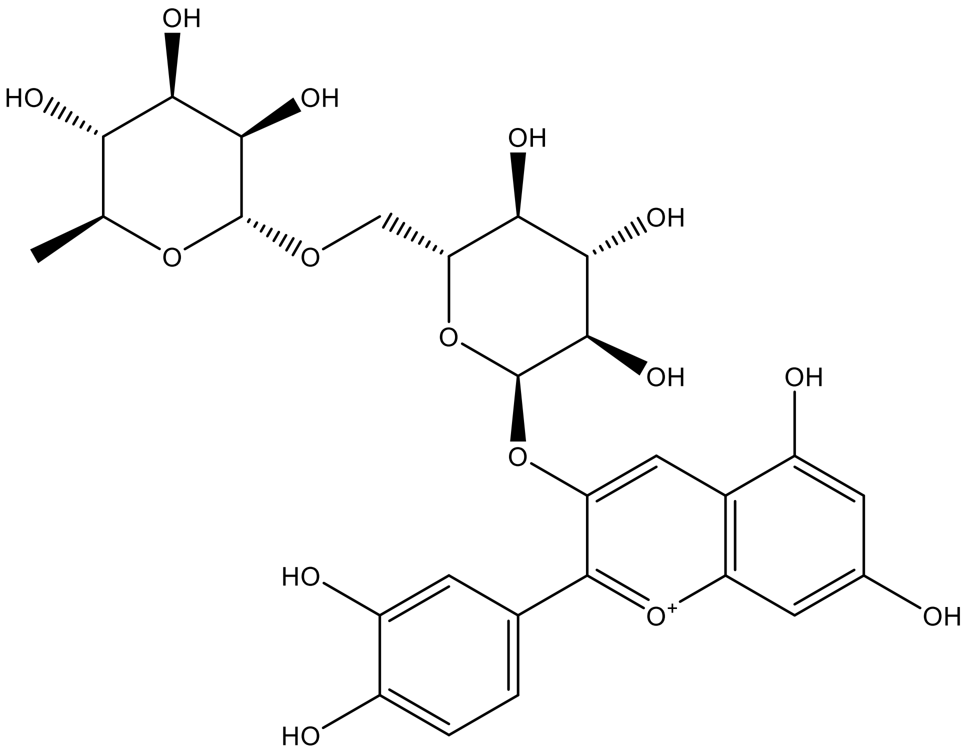

Cyanidin 3-O-rutinoside;

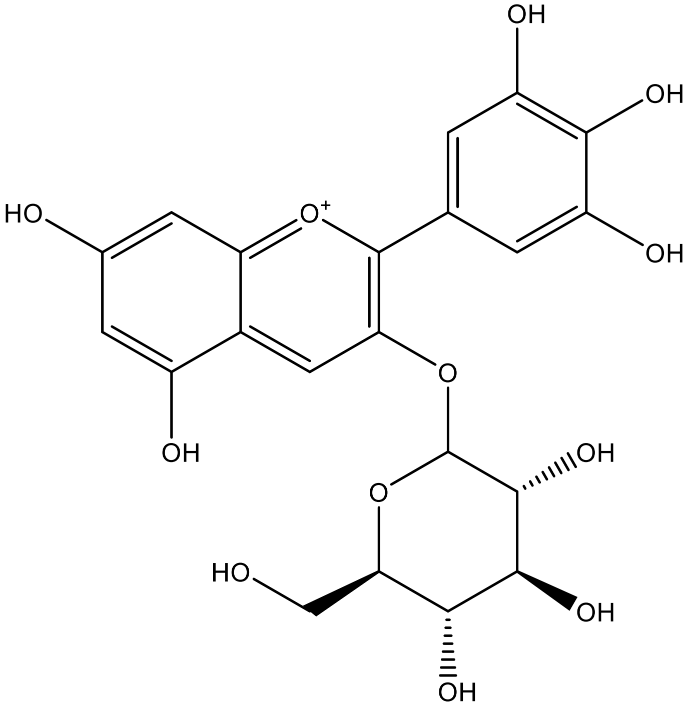

Delphinidin-3-O-glucoside

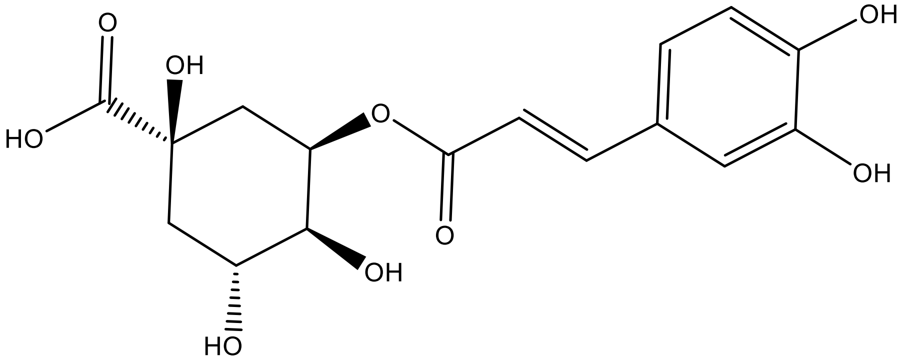

5-caffeoylquinic acid (Neochlorogenic acid)

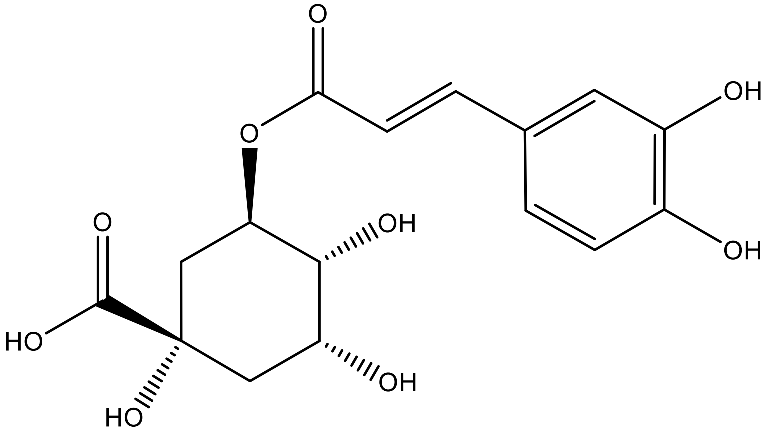

Chlorogenic acid

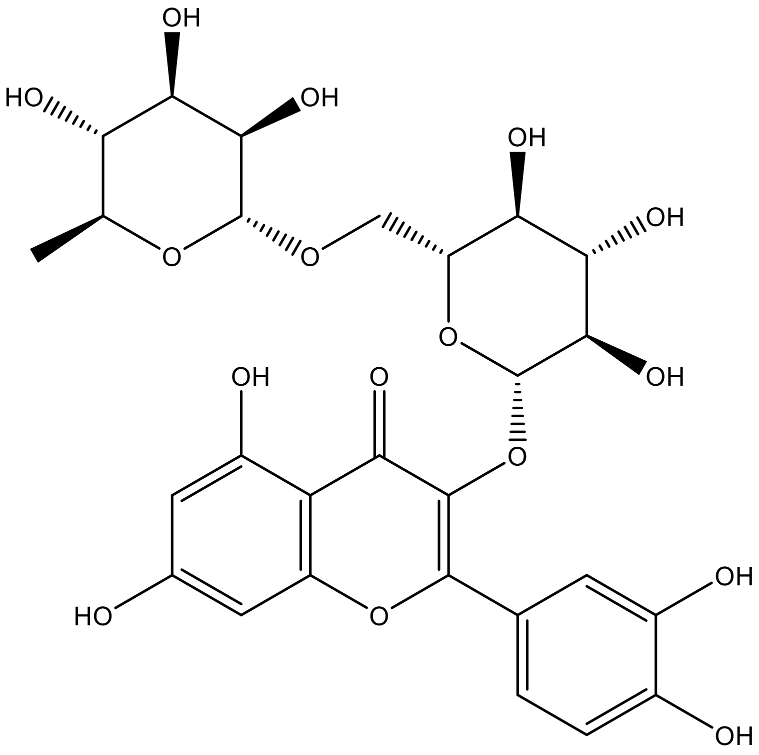

Quercetin-3-O-rutinoside

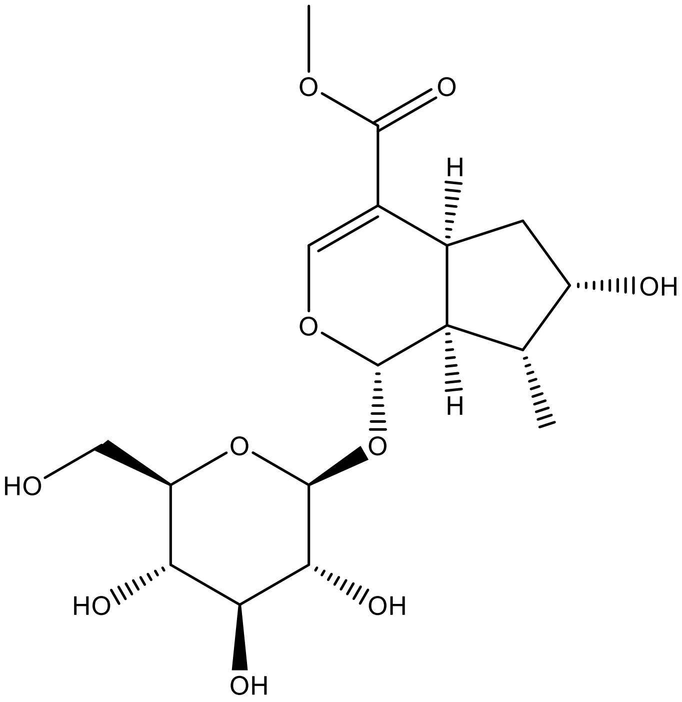

Loganin

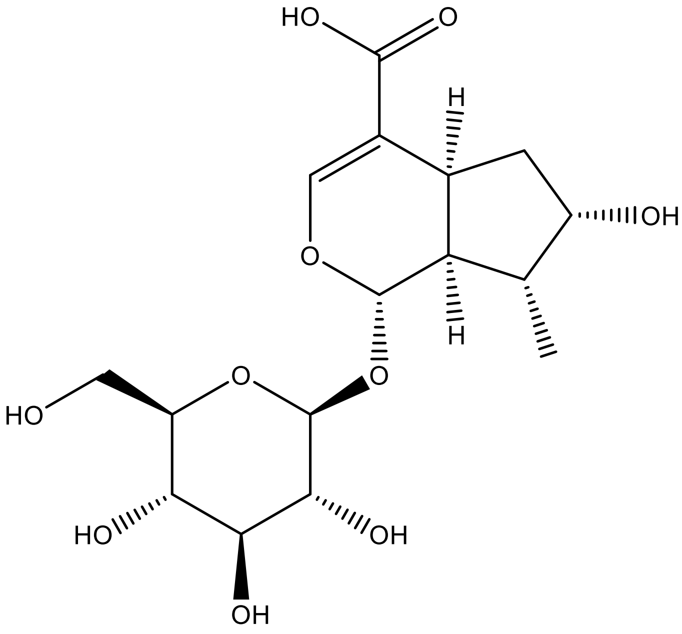

Loganic acid

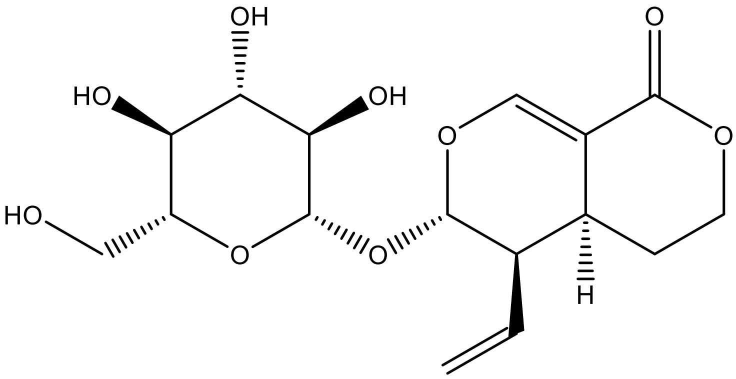

Sweroside

Figure S4. Chemical structures of main compounds found in AKB preparation.
