# Supplementary material for: Standardized Berry Extract Improves Selected Visual Function Outcomes in Presbyopia: A Randomized, Double-Blind, Placebo-Controlled Crossover Trial with Exploratory Biomarker Analysis
Source: Nutrients. 2026 Mar 23;18(6):1016. doi: 10.3390/nu18061016 (PMC13028795; doi:10.3390/nu18061016)
Supplement: Supplementary file 1 [file nutrients-18-01016-s001.zip › Table S1.pdf]

Table S1 Anthocyanins, phenolic acids, iridoids, and flavonols content (mg/100 g fw) of the extract AKB by HPLC.

No.	Compound	Content (g/100 g)
	**ANTHOCYANINS**	
1	Cyanidin 3-O-galactoside	12.610
	Delphinidin 3-O-arabinoside	
2	Cyanidin 3-O-glucoside	9.425
3	Cyanidin 3-O-rutinoside	
	Cyanidin 3-O-arabinoside	6.289
4	Cyanidin 3,5-O-diglucoside	0.990
	Delphinidin 3-O-galactoside	
5	Delphinidin 3-O-glucoside	0.742
6		0.627
	Cyanidin 3-O-xyloside	
7	Peonidin 3-O-glucoside	
	Malvidin 3-O-galactoside	0.549
8	Peonidin 3-O-arabinoside	
	Malvidin 3-O-glucoside	0.409
9	Malvidin 3-O-arabinoside	0.183
10	Peonidin 3-O-galactoside	
	Petunidin 3-O-arabinoside	0.182
11	Other anthocyanins	0.097
**Total anthocyanins**		**32.102**
	**PHENOLIC ACIDS**	
12	5-Caffeoylquinic acid	3.037
13	3-Caffeoylquinic acid	1.940
14	*p*-coumaric acid derivative	1.125
15	Coumaroyl iridoid glycosides	0.658
16	Dicaffeoylquinic acid	0.320
17	*p*-Coumaric acid derivative	0.216
18	Other phenolic acids	0.027
**Total phenolic acids**		**7.322**
	**IRIDOIDS**	
19	Loganin	2.946
	Sweroside	
20	loganic acid	1.390
21	Loganin derivative	0.672
22	Loganic acid derivative	0.522
**Total iridoids**		**5.529**
	**FLAVONOIDS**	
23	Quercetin 3-O-galctoside	
	Quercetin 3-O-glucoside	0.813
24	Quercetin 3-O-rutinoside	0.813
25	Quercetin 3-O-glucuronide	
	Quercetin 3-O-robinobioside	
	Quercetin 3-O-pentoside	0.243
26	Quercetin 3-O-vicianoside	0.225
27	Quercetin-dihexoside 2	0.178
28	Quercetin-dihexoside 1	0.046
29	Isorhamnetin rhamnosylhexoside 1	0.026
30	Isorhamnetin 3-O-vicianoside	0.024
31	Isorhamnetin pentosylhexoside	0.014
32	Isorhamnetin rhamnosylhexoside 2	0.010
33	Myricetin	0.003
**Total flavonoids**		**2.240**
